# Biomarkers for patients with Wilms tumor: a review

**DOI:** 10.3389/fonc.2023.1137346

**Published:** 2023-07-24

**Authors:** Hongfeng Zheng, Jiangui Liu, Xiuwu Pan, Xingang Cui

**Affiliations:** Department of Urology, Xinhua Hospital, School of Medicine, Shanghai Jiao Tong University, Shanghai, China

**Keywords:** Wilms tumor, genetic abnormalities, biomarkers, stratification system, liquid biopsy

## Abstract

Wilms tumor, originating from aberrant fetal nephrogenesis, is the most common renal malignancy in childhood. The overall survival of children is approximately 90%. Although existing risk-stratification systems are helpful in identifying patients with poor prognosis, the recurrence rate of Wilms tumors remains as high as 15%. To resolve this clinical problem, diverse studies on the occurrence and progression of the disease have been conducted, and the results are encouraging. A series of molecular biomarkers have been identified with further studies on the mechanism of tumorigenesis. Some of these show prognostic value and have been introduced into clinical practice. Identification of these biomarkers can supplement the existing risk-stratification systems. In the future, more biomarkers will be discovered, and more studies are required to validate their roles in improving the detection rate of occurrence or recurrence of Wilms tumor and to enhance clinical outcomes.

## 1 Introduction of Wilms tumor

Wilms tumor (WT), also known as nephroblastoma, is the most common renal malignancy in childhood, accounting for approximately 90% of all renal tumors in children. Approximately 95% of patients with WT are under 10 years of age (1). Current standardized diagnostic and therapeutic procedures have made it possible to cure nearly 90% of children with WT. According to the International Society of Pediatric Oncology (SIOP) report (2), the two-year event-free survival (EFS) and overall survival (OS) were 87% and 93%, respectively in children with WT who received the SIOP-2001 protocol with preoperative chemotherapy, while the Children’s Oncology Group (COG) trials report (3) similar results, in which patients received direct operation.

However, postoperative recurrence and high-risk tumors remain formidable clinical challenges. Recurrence rate in WT is approximately 15% of children and is positively correlated with histological risk (2, 4, 5). The anaplastic subtype is the most common histological type in Wilms tumor, which is associated with poorer outcomes (6, 7). Risk stratification systems have been developed to assess clinical outcomes by stratifying tumors at different risk levels. Both the SIOP and COG protocols recognize tumor stage, histology, and volume as prognostic factors to divide patients into subgroups and formulate postoperative therapeutic strategies. In addition, the significance of genetic aberrations is underlined by COG, knowing that a gain of 1q leads to a high risk of relapse and death. With the identification of more WT-associated genes and proteins (4), the relationship between these biomarkers and clinical outcomes has also been gradually disclosed.

In this review article, we begin with the relationship between nephrogenesis and tumorigenesis. We focused on some WT-related genetic abnormalities, briefly overview their pathophysiological mechanism in tumorigenesis, and identify their potential clinical value in WT (**Table 1**). We then discuss copy number variations mentioned in the COG stratification system, which are regarded as prognostic factors for assessing tumor recurrence and extra mortality in a particular cohort. We will also introduce some lncRNA-related studies on WT. Finally, as liquid biopsy is a hot topic in cancer research, we summarized relevant studies and discussed how liquid biopsy was applied to improve WT diagnosis. Although many of these biomarkers are limited by additional factors such as tumor histology, tumor stage, and therapeutic regimens, they have potential value in the diagnosis, prognostic prediction, and therapeutic assessment of patients with WT.

**Table 1:** Genetic abnormorlities in Wilms tumor.

Biomarker	Clinicopathological associations	Refs
*WT1*	**·** Germline mutations relates to various predisposition syndromes (WAGR syndrome, Denys–Drash syndrome, Frasier syndrome) **·** Associated with stromal histology	4, 8–11
*IGF2*	**·** Associated with PLNRs **·** Associated with Beckwith-Wiedemann syndrome	12
*SIX1/SIX2*	**·** Possible reduced relapse-free and overall survival when combined with miRNAPG	13, 14
miRNAPGs	**·** *DIS3L2* mutation is associated with Perlman syndrome **·** Possible reduced relapse-free and overall survival when combined with *SIX1/SIX2*	13–15
*TRIM28*	**·** Associated with epithelial histology	16, 17
*TP53*	**·** Associated with reduced relapse-free and overall survival **·** Associated with diffuse anaplastic histology	18, 19
*MYCN*	**·** Associated with reduced relapse-free and overall survival **·** Associated with anaplastic histology	20, 21
1q gain	**·** Associated with reduced relapse-free and overall survival in both COG and SIOP studies	22, 23
LOH at 1p/16q	**·** Associated with reduced relapse-free and overall survival in COG studies	24, 25
LOH at 11p15	**·** Associated with increased motality in VLRWT group in COG studies	26, 27

VLRWT: very low risk Wilms tumor (patient age<2 years, stage I, favorable histology, and tumor volume <550g).

## 2 Fetal nephrogenesis and Wilms tumorigenesis

**Figure 1:**
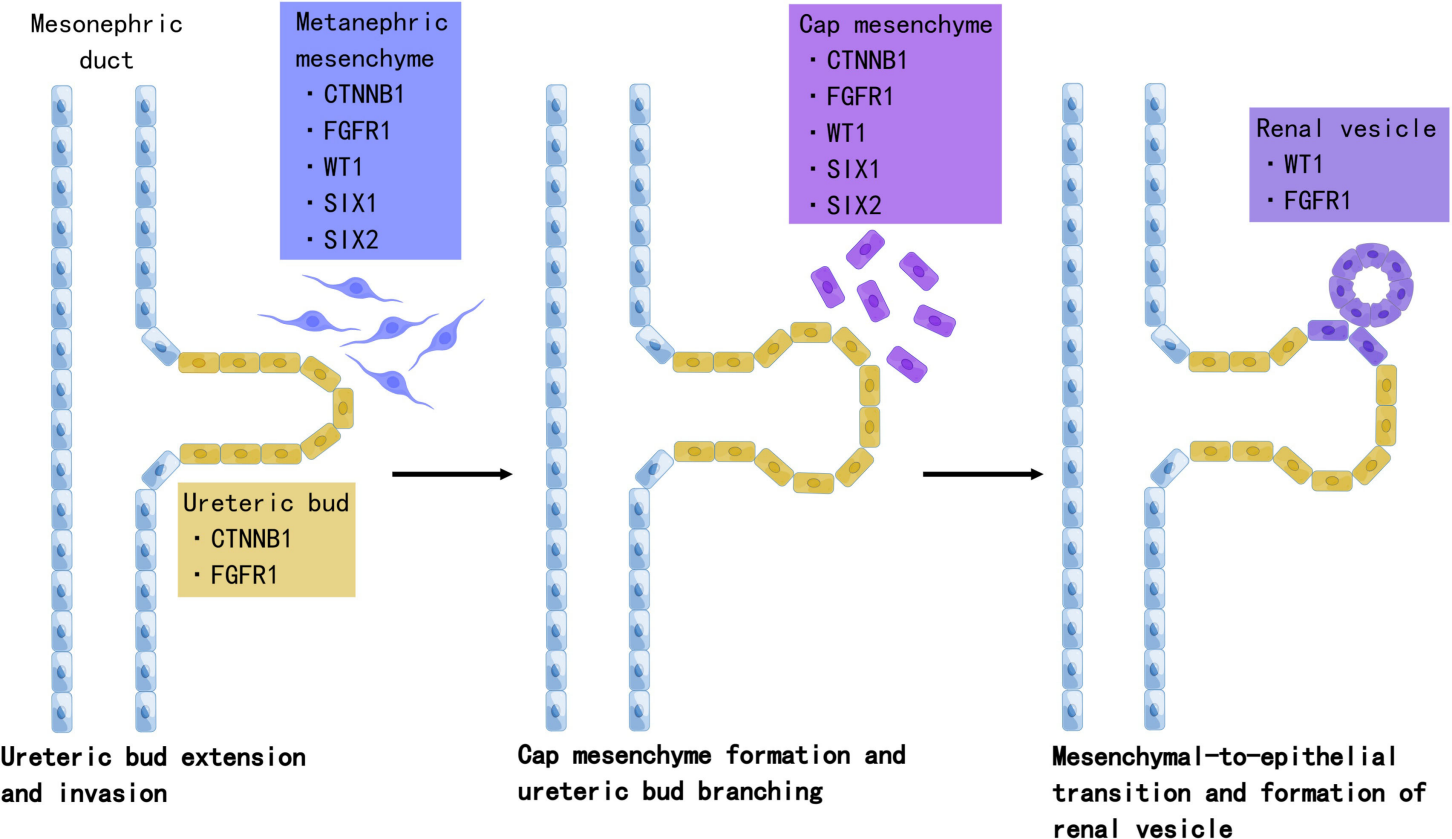
The role of Wilms tumor genes in nephrogenesis. Development of the definitive kidney starts around the fifth week of gestation. Several genes are involved in this process. *WT1* is a key regulator of the entire process, including the development of the metanephric mesenchyme to the cap mesenchyme and renal vesicles. *SIX2* maintains the population of mesenchymal progenitors in an undifferentiated state. Together, *WT1*, *SIX2*, and *CTNNB1* function to facilitate the FGFR pathway. *FGFR1* plays an important role in nephron progenitor cell survival, branching of ureteric buds, and elongation of primitive renal vesicles into comma- and S-shaped bodies that eventually form mature nephrons. Mutations in these genes have been associated with Wilms tumorigenesis. Created by Figdraw.

Accumulating evidence suggests that Wilms tumor originates from aberrant fetal renal development, which evolves into the definitive human kidney and originates from the ureteric bud and metanephrogenic tissue during the fifth week of embryonic development (28). The ureteric bud sprouts from the mesonephric duct branch and invades the metanephric mesenchyme. Under ureteric bud induction, mesenchymal cells condense and undergo mesenchymal-to-epithelial transition (MET), leading to renal vesicles. The ureteric bud and its branches eventually form the collecting duct system, while renal vesicle polarization and elongation form the proximal and distal tubules and loops of Henle. In this process, a complex network of genes controls the balance between self-renewal and differentiation (**Figure 1**).

In WT mice, the process of nephrogenesis can be disrupted at different levels, leading to incomplete differentiation arrest of renal progenitor cells. Thus, WTs are often called the tri-phasic type, because they comprise blastemal, stromal, and epithelial cells, which correlate with cap mesenchyme, uninduced metanephric mesenchyme, and renal epithelial cells, respectively (29). Single-cell transcriptomes in 2018 revealed the relationship between WTs and fetal developing nephron populations, supporting the hypothesis that Wilms tumor is closely linked to stalled renal organogenesis (30). We selected WT genes and discussed their relationship with nephrogenesis and tumorigenesis.

## 3 Genetic abnormalities in WT

### 3.1 Wilms tumor gene 1

*WT1* was the first gene implicated in Wilms tumorigenesis (31). *WT1* encodes an important transcription factor that regulates over 100 genes and is involved in all stages of fetal kidney development (32, 33). In homozygous *WT1* knock-out mice, the development of the metanephric kidney failed (34). Germline *WT1* abnormalities contribute to several WT-associated predisposition syndromes. One of the most common syndromes is WAGR syndrome, which is characterized by Wilms tumor, aniridia, genitourinary anomalies, and a range of developmental delays (WAGR). WAGR is caused by microdeletions at 11p13, including *WT1* deletion and adjacent *PAX6*. Denys–Drash Syndrome (DDS) underlay by*WT1* missense mutation is characterized by ambiguous genitalia and nephropathy secondary to diffuse mesangial sclerosis (8, 9). Moreover, mutations alter the balance of *WT1* splice isoforms, resulting in Frasier Syndrome, which carries the risk of gonadoblastoma and focal glomerulosclerosis (10). The relationship between predisposing genetic conditions and tumor relapse has been reported in previous studies. Both COG and SIOP studies (11, 35) reported that a higher relapse rate was not observed in patients with WAGR than in patients with non-syndromic WT patients, excluding the effect of metachronous tumors. Besides, somatic *WT1* mutations were found in 10%–20% WT patients, without showing independent prognostic value (4, 36).

### 3.2 Insulin-like growth factor 2

Abnormal methylation at 11p15 is the most common genomic change found in the WT, and the *IGF2/H19* domain was detected in this chromosomal region (37). *IGF2* encodes an embryonal growth factor and is regulated by a non-coding RNA transcribed by *H19*. IGF pathway is overactivated by the biallelic expression of *IGF2*, which results from *H19* hypermethylation and subsequent loss of imprinting of *IGF2* (38, 39). During nephrogenesis, perilobar nephrogenic rests (PLNR) are associated with biallelic expression of *IGF2*, which is considered an early event in tumorigenesis (12). Multiple germline changes at 11p15, including epimutation of *H19* or loss of heterozygosity at *IGF2*, are responsible for Beckwith–Wiedemann syndrome, which is susceptible to embryonal tumors, including WT (40). Coorens et al. (41) observed that hypermethylation of *H19* with subsequent overexpression of *IGF2* was directly associated with clonal nephrogenesis and the development of Wilms tumor in a cohort of 23 patients with WT. Although the prognostic value of *IGF2/H19* was not explored, the authors suggested that the relationship between clonal nephrogenesis and formation of WT should be emphasized, which could be utilized to guide the surveillance schedule of patients with WT.

### 3.3 SIX1/SIX2

Several studies (13, 14) have identified *SIX1* and *SIX2* as WT-specific oncogenes, both of which are associated with the blastemal subtype, another high-risk histology in the SIOP protocols. *SIX1* and *SIX2* are key regulators of nephrogenesis. Expression of cell cycle genes was found to be upregulated in *SIX1*- and *SIX2*-mutant WT mice, and loss of *SIX1* resulted in mesenchymal apoptosis in *SIX1*-knockout mice, while *SIX2* activity maintained the number of nephrogenic progenitors in undifferentiated blastemal tissues (42, 43). In addition, *SIX2* overactivation in a renal cell line increased the percentage of cells in the S-phase (13, 14). Walz et al. reported that patients with combined *SIX1/SIX2* and microRNA processing genes (miRNAPGs) mutations had a significant higher relapse rate (80%, p = 0.001)and a higher mortality (40%), though the *SIX1/SIX2* and miRNAPGs variants alone did not show bad outcomes (14).

### 3.4 microRNA processing genes and microRNA

**Figure 2:**
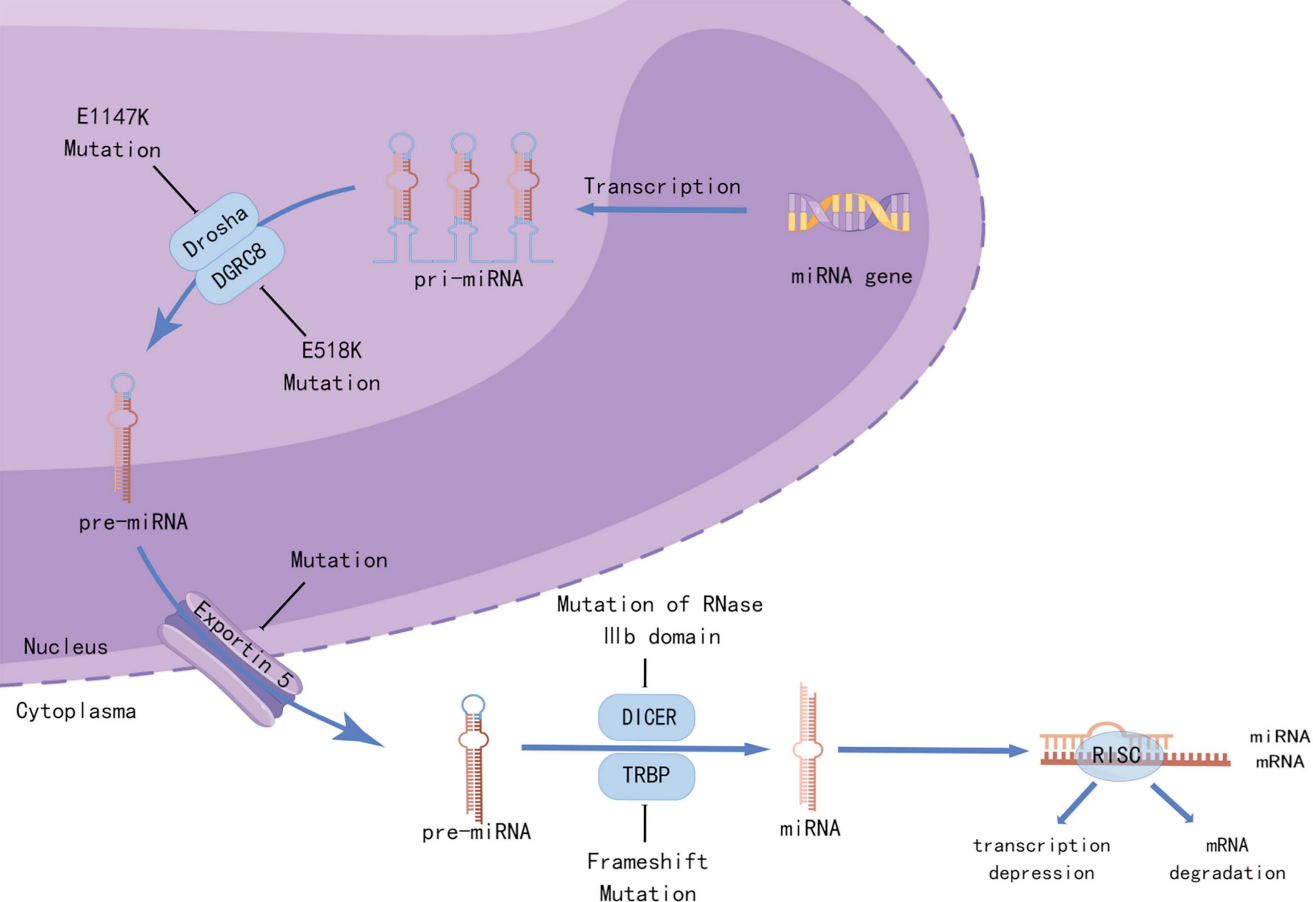
Mutations in miRNA-processing genes lead to aberrant miRNA biogenesis. Recurrent mutations in the metal-binding (Mg2+) residue of the RNase IIIb domain of DROSHA (E1147K) or the doublestranded RNA-binding domain of DGRC8 (E518K) disrupted the cleavage of pri-miRNAs into pre-miRNAs. Mutations in *XPO5* (encoding exportin 5) prevent pre-miRNA export, resulting in premiRNA accumulation in the nucleus. Frameshift mutations in *TARBP2* (encoding TRBP) and those affecting the RNase IIIb domain of DICER1 can disrupt the processing of pre-miRNAs into mature miRNAs. Created by Figdraw.

Whole-genome and whole-exosome sequencing of WT have been used to identify unique mutations in microRNA processing genes (miRNAPGs), including *DROSHA*, *DICER1*, *DGCR8*, *XPO5*, and *TARBP2* (13–15), which lead to impaired miRNA biogenesis (**Figure 2**). Approximately 33% of the WTs examined carried mutations in miRNAPGs (44). Combined mutations in both *SIX1/SIX2* and miRNAPG resulted in poorer outcomes in a COG study (14). As microRNAs (miRNAs) are critical regulators of kidney morphogenesis by modulating diverse biological processes in different renal cell lineages, mutations in miRNAPGs lead to the downregulation of important microRNAs (miRNAs). Global downregulation of mature *let7* family miRNAs occurs in *DROSHA* mutants, resulting in failure of epithelial differentiation (15, 44). The RNA-binding protein Lin28 suppresses the processing of *let7* miRNA, and the balance between them controls the timing of nephrogenesis in mice (45). Overexpression of *LIN28* inhibits the differentiation of nephrogenic progenitors, thus causing neoplastic transformation, which is similar to the situation in human WT (46). Copy number gain of *LIN28B* and loss of *let7* were observed in 25% and 46% of the human WT, respectively (20). *DIS3L2*, which encodes an exoribonuclease responsible for degrading preprocessed forms of *let7*, was found to be mutated in Perlman syndrome, which is characterized by macrosomia, polyhydramnios, facial dysmorphology, renal dysplasia, and predisposition to WT (47). The *miR-200* family, which is key to the mesenchymal-to-epithelial transition, was also found to be downregulated as a result of miRNAPG mutations and is associated with an undifferentiated blastemal histology (14). A review by Cerqueira et al. (48) summarized multiple studies and found that aberrant expression of specific miRNAs was correlated with the etiology of WT. These miRNAs not only function as oncogenes but also as tumor suppressors in WT development.

Notably, the expression levels of some miRNAs were associated with clinical outcomes. One study (49) reported the upregulation of 14 miRNAs in the serum of patients with WT. They found that the expression levels of *miR-110-5p* and *miR-130-3p* could be used to differentiate WT children from healthy children. Apart from their potential predictive value, there are several additional reasons to support miRNAs as detectable biomarkers. MicroRNAs are widely distributed in various organisms. Apart from their intracellular location, their distribution in body fluids makes it non-invasive to capture sufficient samples (50). In addition, circulating miRNAs are conjugated to other macromolecules, thus facilitating their stable storage (51, 52). However, hurdles also exist and should be overcome using standardized methodologies for the purification and analysis of samples. In addition, studies with large sample sizes are required. In conclusion, miRNAs have great potential as biomarkers because of their unique biological features and potential clinicopathological value.

### 3.5 TRIM28

*TRIM28*, a classic WT tumor suppressor gene, is predisposed to familial or non-familial WT with germline mutations (16, 17). WT with *TRIM28* mutations is associated with epithelial histology, which shows a better prognosis. Hol et al. (16) reviewed all previously reported cases, and follow-up data were available for 13 patients with germline pathogenic variants in *TRIM28* and found that no relapse occurred in any of these patients. Although the epithelial histological type has been reported to be associated with good outcomes (53), whether the prognostic value of *TRIM28* mutations is independent of epithelial histology remains to be validated. As *TRIM28* germline mutation can be simply detected by immunohistochemistry using anti-KAP1 antibody in WT patients (17), it can be used to recognize other young family members predisposed to tumors.

### 3.6 TP53

Somatic mutations in *TP53* are one of the most frequent alternations in human cancers, and germline mutations are the underlying cause of Li–Fraumeni syndrome, which predisposes to a range of cancers (54). In patients with WTs, *TP53* mutations is frequently detected in the anaplastic subtype, especially in diffuse anaplastic Wilms tumor (DAWT) (7, 55). Ooms et al. (18) reported *TP53* mutations in 57 (48%) of 118 DAWT cases, 13 (11%) cases of copy loss without mutation, and 48 (41%) cases lacking both. In contrast to those with *TP53* abnormalities, DAWTs with *TP53*-wide-type indicate lower relapse and death rates in stage III/IV patients. As diffuse anaplasia correlates with poor outcomes, *TP53* status further improves risk stratification in DAWT, meaning that patients with *TP53* mutations should receive more intensive treatment (19). In view of the correlation between *TP53* mutations and DAWT, early identification of this high-risk histological subtype could be done by detecting *TP53* mutations in circulating tumor DNA to determine whether intensive preoperative chemotherapy should be provided (56).

*TP53* mutations are not limited to anaplasia. In blastemal and some intermediate-risk histology subtypes, *TP53* mutations were also observed to be correlated with a high risk of death (13). Wegert et al. (55) suggested that *TP53* might play a driving role in the histological progression of WTs, as partial features of anaplasia were found in some blastemal tumors. *TP53*-screening should be launched at an early stage, not only to identify anaplasia before surgery, but also to access tumor progression. As intratumoral heterogeneity may cause trouble, multiple sampling is needed by applying liquid biopsies to capture adequate tumor circulating DNA and harbored *TP53* mutations. We further discuss circulating tumor DNA in *Section 5*.

### 3.7 MYCN

Mutations in *MYCN* have also been associated with high-risk anaplastic histology. Williams et al. (21) reported that 30.4% (7/23) samples had *MYCN* gain in the diffuse anaplastic subtype compared to 11.2% (30/269) in other subtypes, indicating a significant association (p = 0.0159). In this study, *MYCN* gain was found to be correlated with poorer relapse-free survival and OS in cases of all histology and in cases with diffuse anaplasia. Interestingly, *MYCN* mutations are three times less frequent in DAWTs in the COG cohort (20). Although this skewing did not reach statistical significance, it agrees with the conclusion of most recent studies that *MYCN* mutations have prognostic value, whether anaplastic or not.

## 4 Long noncoding RNAs in WT

Long noncoding RNAs (lncRNAs) are a large group of nonprotein-coding RNAs consisting of more than 200 nucleotides. lncRNAs are involved in many biological processes, including gene silencing, gene imprinting, RNA interference, and protein translation and modification (57–59). Disruption of lncRNA expression is intrinsically linked to a variety of diseases, including cancer (60). The role of lncRNAs in WT has not been fully elucidated, although relatively few studies have been conducted in the recent years. For example, *WT1*, the most prominent WT relative gene, is directly or indirectly regulated by lncRNAs. WT1 antisense RNA (WT1-AS), originating from the intron region of *WT1*, can bind to WT1 mRNA and regulate WT1 protein expression by RNA–RNA interactions (61). Recent studies have demonstrated that WT1-AS plays a significant role in many tumors; however, its roles vary among different tumors. Dallosso et al. found high expression levels of WT1-AS in WT (62); however, its relationship with clinical outcomes and prognosis has not been clarified. However, the specific mechanisms of action need to be elucidated.

According to the competing endogenous RNA (ceRNA) theory, lncRNAs regulate the expression of target genes by adsorbing miRNAs (63). To further explore the role of lncRNAs in tumorigenesis, several studies have established ceRNA networks to identify the potential lncRNAs as much as possible involved in WT. Wang et al. (64) constructed a lncRNA–miRNA–mRNA ceRNA network consisting of 32 lncRNAs, 14 miRNAs, and 158 mRNAs. Subsequently, three lncRNAs, three miRNAs, and 17 mRNAs were found to be associated with OS. Of the three lncRNAs, MYCN opposite strand (*MYCNOS*), deleted in lymphocytic 2 (*DLEU2*), was highly expressed in the late stages of WT and correlated with poorer OS, whereas upregulation of chromosome 8 open reading frame 31 (C9orf31) in the early stage may play a protective role. Similar results regarding *MYCNOS* and *DEUL2* have also been reported in other studies on neuroblastoma, laryngeal carcinoma, and leukemia (65–67). In addition to the prognostic correlation, some studies have established predictive survival models. Liu et al. (68) constructed three models based on survival-associated RNAs (lncRNAs, miRNAs, and mRNAs) from primary solid WT tissue and AUC values of these models were all greater than 0.7, denoting excellent model performance. Although significant results have been obtained, more applicable predictive models must be built based on multicenter data and various pathological tissues.

## 5 Copy number variations in stratification system

Both the SIOP and COG use stages and histological subtypes were used to stratify risks in postoperative patients. Since 2005, COG has included a molecular marker in risk stratification, recommending that children whose tumors have loss of heterozygosity (LOH) for alleles spanning chromosomes 1p and 16q should receive more intensive chemotherapy (26, 69). In addition, 1q gain and LOH at 11p15 showed clinical value in a particular subgroup of patients. Although the precise mechanism of oncogenesis in tumors with these copy number variations remains unclear, their association with relapse and death is important in clinical practice.

### 5.1 1q gain

The gain of chromosome arm 1q is a significant factor associated with poorer clinical outcomes in terms of reduced OS and shorter EFS in both COG and SIOP-treated patients (22, 23, 70, 71). In COG studies, gain of 1q was detected in 27% of patients with favorable histology WT (FHWT) and showed significance in OS and EFS as a marker independent of tumor stage (22, 23). The COG is planning to incorporate it into risk stratification in the next series of studies. In addition, the SIOP study has recognized 1q gain as a potential prognostic biomarker in WT, and they aimed to further validate its role in the stratification of patients who have received preoperative chemotherapy (70).

### 5.2 LOH at 1p and 16q

According to a previous National Wilms Tumor Study Group (NWTSG) study (69), LOH at 1p only (LOH 1p), 16q only (LOH 16q), and combined 1p and 16q (LOH 1p/16q) was associated with an adverse outcomes in patients with stage I/II favorable-histology WT treated with immediate nephrectomy. In patients with stage III/IV disease, only LOH 1p/16q is associated with an increased risk of relapse and death. Another study (24) reported that in patients with non-anaplastic WT, only LOH 1p had prognostic value, while LOH 16q and LOH 1p/16q did not. Messahel et al. (25) found that LOH 16q and LOH 1p/16q were related to increased risk of relapse and death in patients with favorable histology tumor, whether the patients had received initial therapies or not. LOH 1p and/or LOH 16q appeared to have an independent prognostic effect in the 1q-gain-negative group when patients with or without 1q gain were analyzed separately (23). In summary, LOH 1p, LOH 16q, and LOH 1p/16q have limited but not completely independent prognostic values and are applied in a particular subgroup of patients in COG studies.

In the SIOP study, neither LOH 1p nor LOH 16q, nor LOH 1p/16q can be considered as a single biomarker related to poorer EFS or OS at p = 0.05, whether in the univariate or multivariate analysis (70), which conflicts with the COG observations. The prognostic value of LOH 1p and/or 16q should be further validated in SIOP patients (72).

### 5.3 LOH at 11p15

COG stratification defines a group of patients as a very low-risk subgroup (younger than 2 years, stage I, favorable histology, and tumor volume <550 g), who are at low risk of relapse and only need to undergo direct surgery without adjuvant chemotherapy (26). However, if LOH at 11p15 exists, operation-only treatment is not effective and chemotherapy is indispensable because LOH at 11p15 is associated with a higher rate of relapse (26, 27).

## 6 Liquid biopsy

Tumor biopsies at diagnosis, resection, or relapse are the gold standards for identifying tumor biology, diagnosis, and therapeutic decision-making. However, the shortcomings are also obvious, such as unavoidable trauma caused by puncture or surgery and over-dependence on imaging examination. Solid pediatric tumors are more likely to shed tumor cells, DNA, RNA, or proteins into the blood or urine. Since blood or urine samples are easily available at any time, identification of these tumor markers in body fluids, also known as liquid biopsy, is a better and potential measure to manage tumor patients. Liquid biopsy has unique advantages in that it can screen primary lesions, monitor recurrence, and assess the treatment effect in patients with WT by identifying tumor markers in a real-time manner (73–76).

A signature of 176 circulating miRNAs was diagnostic of WT and could distinguish healthy children (77). *TP53* mutations in circulating tumor DNA (ctDNA) detected by liquid biopsy can help to identify DAWT at an early stage, one of the most invasive subtypes of WT (56). A COG trial (74) reported the detection of ctDNA in the serum of 41/50 (82%) and urine of 13/50 (26%) patients with stage III/IV disease, and the agreement between serum ctDNA and tumor sequencing results was highly significant. Detectable ctDNAs include CNVs (1q gain, LOH at 1p and/or 16q) and single-nucleotide variants (*WT1*, *CTNNB1*, *MYCN*, and *TP53*). OS and EFS in patients with detectable ctDNA in serum were poorer than those in patients without (positive vs. negative group: 82.79% vs. 100% for OS, 80.41% vs. 100% for 4 years-EFS), whereas the discrimination effect of urine ctDNA was not significant between the two groups (positive group vs. negative group: 76.92% vs. 91.43% for OS, 76.92% vs. 88.57% for 4 years-EFS) (74). Moreover, circulating miRNA detection can be used to differentiate WT from other pediatric tumors (78). In other cancers, ctDNA has been shown to capture the presence of subclonal heterogeneity better than solitary biopsies (79–81), which provides a reference for WT management.

In addition to nucleic acid detection, protein biomarkers can be profiled using high-resolution mass spectrometry (HRMS) proteomics of urinary specimens. Previous studies (82–84) reported that neuron-specific enolase, basic fibroblast growth factor (bFGF), and hyaluronidase have been reported to be enriched in the urine of patients with Wilms tumor. In addition, bFGF overexpression in urine is related to the WT stage and has prognostic value (83). Ortiz et al. (85) reported that prohibitin in FHWTs acted as a prognostic marker in tumor relapse and a cutoff threshold of 998 ng/ml was a predictor of recurrence, especially recurrence in the abdomen (AUC: 0.78 for all recurrence, 0.96 for abdominal recurrence). DACT2 and DAD1 proteins were only mentioned briefly in their study and no further validated. The review by Coppes et al. (86) mentioned “paraneoplastic syndromes” in WT and several associated factors, including neuron-specific enolase (NSE), hyaluronic acid (HA), hyaluronic acid-stimulating activity (HSA), and hyaluronidase, all of which may predict recurrence or evaluate the therapeutic effect. SIOP aims to establish biobanks by collecting serial blood and urine samples, as well as tumor and germline material at diagnosis and specific time points during treatment for international collaborative studies (87). Similarly, the COG study also utilized liquid biopsy to test the potential benefits of diagnostics, monitoring of therapy, and detection of residual disease (88).

## 7 Conclusion

Remarkable progress has been made in the early detection and management of cancer progression and recurrence owing to advances in risk stratification systems, treatment, and follow-up protocols. As tumor stage and histological subtype have clearly shown relevant prognostic value, the introduction of Wilms tumor biomarkers has further completed the risk stratification systems, the targeting capability of the treatment measures, and follow-up plans. Among various biomarkers, copy number variations, such as 1p/16q LOH have displayed significant prognostic value and have been successfully applied in COG protocols. *TP53* and *MYCN* mutations have confirmed clinicopathological associations, showing promising application potential. Others, especially miRNAs and proteins, also exhibited their potential as novel tumor biomarkers in the future due to their close association with tumorigenesis. In addition to prognostic value, alterations in some biomarkers are early events in WT tumorigenesis, showing promising perspectives in predicting tumorigenesis before routine laboratory tests and imaging examinations. Because blood and urine samples are easily available, all biomarkers can be monitored dynamically. These measures will greatly improve the primary or secondary tumor screening rate and shorten the window period.

With further research on the mechanism of tumor occurrence and progression, the future objectives of research should focus on saving patients with relapsed and refractory Wilms tumor, while, on the other hand, identifying children with excellent prognosis to release their therapeutic burden. Future studies should continue to discover more biomarkers, clarify their underlying biological mechanisms, and define their predictive and prognostic value for the benefit of WT patients.

## Author contributions

All authors had full access to all the data in the study and take responsibility for the integrity of the literature. All authors were involved in critical revision of the manuscript for important intellectual content. All authors have read and agreed to the published version of the manuscript.
